# Long-Term Adverse Effects of Oxidative Stress on Rat Epididymis and Spermatozoa

**DOI:** 10.3390/antiox9020170

**Published:** 2020-02-19

**Authors:** Pei You Wu, Eleonora Scarlata, Cristian O’Flaherty

**Affiliations:** 1 Department of Surgery (Urology Division), McGill University, Montréal, QC H4A 3J1, Canada; pei.y.wu@mail.mcgill.ca (P.Y.W.); eleonora.scarlata@mail.mcgill.ca (E.S.); 2 Department of Pharmacology and Therapeutics, McGill University, Montréal, QC H3G 1Y6, Canada; 3 The Research Institute, McGill University Health Centre, Montréal, QC H4A 3J1, Canada

**Keywords:** reactive oxygen species, testis, antioxidant enzymes, peroxiredoxins, sperm maturation

## Abstract

Oxidative stress is a common culprit of several conditions associated with male fertility. High levels of reactive oxygen species (ROS) promote impairment of sperm quality mainly by decreasing motility and increasing the levels of DNA oxidation. Oxidative stress is a common feature of environmental pollutants, chemotherapy and other chemicals, smoke, toxins, radiation, and diseases that can have negative effects on fertility. Peroxiredoxins (PRDXs) are antioxidant enzymes associated with the protection of mammalian spermatozoa against oxidative stress and the regulation of sperm viability and capacitation. In the present study, we aimed to determine the long-term effects of oxidative stress in the testis, epididymis and spermatozoa using the rat model. Adult male rats were treated with tert-butyl hydroperoxide (t-BHP) or saline (control group), and reproductive organs and spermatozoa were collected at 3, 6, and 9 weeks after the end of treatment. We determined sperm DNA oxidation and motility, and levels of lipid peroxidation and protein expression of antioxidant enzymes in epididymis and testis. We observed that cauda epididymal spermatozoa displayed low motility and high DNA oxidation levels at all times. Lipid peroxidation was higher in caput and cauda epididymis of treated rats at 3 and 6 weeks but was similar to control levels at 9 weeks. PRDX6 was upregulated in the epididymis due to t-BHP; PRDX1 and catalase, although not significant, followed similar trend of increase. Testis of treated rats did not show signs of oxidative stress nor upregulation of antioxidant enzymes. We concluded that t-BHP-dependent oxidative stress promoted long-term changes in the epididymis and maturing spermatozoa that result in the impairment of sperm quality.

## 1. Introduction

Infertility is a concerning pathophysiological condition that affects about 16% of couples worldwide, and approximately half of the cases are attributable to male factors [1]. Even though the cause for the majority of the male infertile cases is unknown, oxidative stress caused by a high amount of reactive oxygen species (ROS) has been observed in 30–80% of infertile patients [2,3]. Oxidative stress is a common feature associated to environmental pollutants, chemotherapeutic agents and other drugs, smoke, toxins, radiation, and diseases such as many types of cancer that have negative effects on fertility [4,5,6,7,8]. Even though low ROS levels are required for the acquisition of fertilization ability by the spermatozoon [9], oxidative stress damages spermatozoa by reducing motility, and increasing levels of DNA, protein oxidation and lipid peroxidation [10,11,12]. As occurring in somatic cells, this oxidative stress observed in spermatozoa is the result of an imbalance between the antioxidant defense system and the endogenous generation of ROS. 

Peroxiredoxins (PRDXs) are a family of antioxidant enzymes that are highly expressed from yeast to humans. They are peroxidases that do not require co-factors such as heme group or selenium and contains one or two cysteine (Cys) residues in their active site which are essential for their antioxidant function [13,14]. PRDXs isoforms are divided into 2-Cys PRDXs (PRDX1-4), atypical PRDX (PRDX5) and 1-Cys PRDX (PRDX6). These enzymes are important antioxidants in spermatozoa that regulate the level of ROS such as peroxides (H_2_O_2_ and organic hydroperoxides) and peroxinitrite (ONOO^−^), to avoid cellular toxicity [14,15]. Studies using the PRDX6 knockout mouse model have shown sub-fertility associated with severe impairment of sperm motility and high levels of lipid peroxidation and sperm DNA damage. Interestingly, this abnormal reproductive phenotype worsens with aging [16,17]. Other antioxidant enzymes that fight against oxidative stress in testis and epididymis are catalase, glutathione peroxidases, and thioredoxins [18,19,20].

Epididymal maturation is a crucial step in the formation of viable and healthy spermatozoa in humans and other mammals [21]. After spermatogenesis, fully formed yet immature and immotile spermatozoa enter into the epididymis, and by the time of exit, they acquire the ability to move and morphological features that optimize their fertilization capacity [21,22]. In addition to sperm maturation, the epididymis also provides essential proteins to spermatozoa via epididymosomes to maintain their cellular functions and to protect them from potential damages such as oxidative stress-dependent injuries [23]. 

We previously reported that spermatozoa from rats challenged with an in vivo oxidative stress using tert-butyl hydroperoxide (t-BHP), and collected 24 h after the treatment, have higher levels of DNA oxidation and lipid peroxidation and displayed poor motility compared to untreated controls [18]. There was a differential expression of PRDX1 and PRDX6 in a different segment of the epididymis of the treated rats. Interestingly, these spermatozoa contain high levels of PRDXs, as an attempt of the epididymis to fight against the oxidative stress established by the treatment [18]. 

In this study, we aimed to elucidate the long-term effect of t-BHP induced oxidative stress on rat reproductive system by assessing the oxidative damage and the expression of significant antioxidants enzymes that fight against hydroperoxides in spermatozoa, epididymis and testis.

## 2. Materials and Methods

### 2.1. Materials

Tert-butyl hydroperoxide (t-BHP), sodium dodecyl sulfate (SDS), phosphotungstic acid, buthylated hydroxytoluene, 2-thiobarbituric acid and malonaldehyde bis(dimethyl acetal), the Bicinchoninic protein determination assay and the anti-α-tubulin were purchased from Sigma-Aldrich Chemical Co. (St. Louis, MO, USA). The following were purchased from Abcam Inc., (Cambridge, MA, USA): rabbit polyclonal anti-PRDX1, mouse monoclonal anti-PRDX6, mouse monoclonal anti-4-Hydroxynonenal (4-HNE), rabbit polyclonal anti-catalase, and 8-hydroxy-deoxyguanosine (8-OHdG). Anti-thioredoxin 1 antibody was purchased from Cell Singaling Thecnology (Danvers, MA, USA), Polyvinylidene fluoride (PVDF) membranes (0.22 µm pore size; Osmonics Inc., Minnetonka, MN, USA), donkey anti-rabbit IgG and goat anti-mouse IgG, both conjugated to horseradish peroxidase (Cedarlane Laboratories Ltd., Hornby, ON, Canada), an enhanced chemiluminescence kit (Lumi-Light; Roche Molecular Biochemicals, Laval, QC, Canada) and radiographic films (Denville Scientific, Inc., Saint-Laurent, QC, Canada) were also used for immunodetection of blotted proteins. Other chemicals were used at the reagent level.

### 2.2. Animals and Treatment

Adult male Sprague Dawley rats (*n* = 24) were randomly distributed in t-BHP and control groups and were treated with 300 µmoles tert-BHP/kg b.w. or saline (control) once a day intraperitoneally for 15 days, respectively as done previously [18]. Treatment with tert-BHP showed to have no effects on the health of rats [18,24]. Animals were euthanized at 3, 6, and 9 weeks post-treatment. These end points correspond to late, middle and early spermatogenesis, respectively [25]. At each given end time, reproductive organs were collected, weighted and kept at −80 °C until use. For sperm motility determination, cauda epididymes were cut one time at the base with a surgical blade and placed in phosphate-buffered saline (PBS; 1 mM KH_2_PO_4_, 10 mM Na_2_HPO_4_, 137 mM NaCl, 2.7 mM KCl, pH 7.4) at 37 °C. Spermatozoa were allowed to swim-out for 10 min and were collected in clean tubes. All procedures with rats (handling, euthanasia, collections of tissues, etc.) were carried out following the regulations of the Canadian Council for Animal Care (CACC) and according the protocol #2009-5656 approved by the Facility Animal Care Committee (FACC) of the Research Institute, McGill University Health Centre.

### 2.3. Testes and Epididymes Homogenates Preparation

Control and t-BHP treated adult male Sprague-Dawley rats’ frozen testis, caput and cauda epididymis were thawed, weighed and homogenized in a glass potter in RIPA buffer containing protease inhibitors. The samples were then sonicated for 20 s at 30% amplitude twice with 20 s intervals with a Sonic Vibracell (Sonics and Materials, Inc., Newtown, CT, USA). The samples were centrifuged at 21,000× *g* for 20 min at 4 °C. The supernatant was extracted, aliquoted and stored at −80 °C.

### 2.4. Sperm Motility and DNA Oxidation Determinations

Sperm motility was assessed by the same observer (CO) using the Olympus BH-2 microscope at 100 magnification with a thermal plate at 37 °C. At least 200 spermatozoa per duplicate were analyzed to determine percentage of total motility in each sample [18]. Sperm DNA oxidation was determined by immunohistochemistry using the anti-8-OHdG antibody as done previously [18]. Briefly, sperm samples were centrifuged at 2000× *g* for 5 min to remove the PBS medium and resuspended in 20 mM phosphate buffer (pH 6.0) with 1 mM EDTA for 5 min. Samples were then centrifuged and resuspended in 50 mM Tris-HCl (pH 7.4), 1% SDS and 40 mM dithiothreitol for 30 min. Final centrifugation of 5 min to replace the mixture with PBS was performed. The sperm PBS solution was smeared on Superfrost Plus slides (Fischer Scientific, Ottawa, ON, Canada) and they were fixed with 100% methanol at 20 °C for 30 min. Slides were incubated with 5% horse serum for 30 min at room temperature, then washed with PBS-T for 5 min and incubated with anti-8-OHdG antibody (1:100) (SMC-155D, StressMarq Biosciences Inc., Victoria, BC, Canada) diluted in 1% horse serum overnight at 4 °C. After a wash with PBS, the samples were incubated with biotinylated horse anti-mouse antibody in 1% BSA and PBS-T for 1 h, washed and finally incubated with Alexa Fluor 555-streptavidin (1:500 in PBS) for 45 min at 20 °C. ProLong Gold antifade with DAPI was added and smears sealed. Slides were analyzed with Zeiss Axiophot fluorescence microscopy (Carl Zeiss, Oberkochen, Germany). Two hundred spermatozoa per slide were counted in duplicate. A positive control was done by incubating spermatozoa with 2 mM H_2_O_2_ for 1 h at 37 °C. The specificity of the antibody was confirmed previously [18].

### 2.5. Testes Histological Analysis, and Sperm Count

Testes were dissected, weighed, and fixed immediately with Bouin fixative for 24 h before processing and embedding in paraffin blocks, and tissues were sectioned (5 μm) and stained with hematoxylin-eosin as previously described [26]. Spermatozoa heads from testis homogenates were counted in a hematocytometer as previously described [27]. 

### 2.6. SDS-PAGE and Immunoblotting 

The bicinchoninic acid assay was performed to determine protein concentration in each tissue homogenate sample. Testis and epididymis tissue samples were mixed in electrophoresis sample buffer supplemented with 100 mM DTT, incubated at 95 °C for 5 min, and then centrifuged at 21,000× *g* for 5 min. Proteins in the supernatant were electrophoresed on 12% polyacrylamide gels and electrotransfered to polyvinylidene difluoride membranes. Then, the membranes were incubated in a solution of skim milk (5%, *w*/*v*) in Tween-containing Tris-buffered saline (TTBS; 20 mM Tris, 0.1% *v*/*v* Tween, pH 7.8) for 30 min followed by the incubation in anti-PRDX-1 (1:10,000), anti-PRDX-6 (1:10,000), anti-catalase (1:1000), anti-4-hydroxynonenal (4HNE) (1:100), anti-Thioredoxin1 (TRX1) (1:500) primary antibodies overnight. To test the specificity, 0.4 μg/mL of anti-PRDX1 was incubated with 2 μg/mL of its antigenic peptide in TBS-T supplemented with 3% BSA for 2 h at room temperature [18]. The absence of non-specific binding was confirmed by the incubation of tissue samples with the secondary antibody (goat anti-mouse or donkey anti-rabbit IgG) only. After being washed with TTBS, the membranes were incubated with goat anti-mouse or donkey anti-rabbit IgG conjugated with horseradish peroxidase (diluted 1:2000 in TTBS) for 45 min at room temperature and washed again with TTBS. The immunoreactive bands were detected using Lumi-Light chemiluminescence kit. Then, the membranes were stripped and re-blotted with an anti-tubulin antibody to determine equal loading. Silver staining was used to determine equal loading in samples under non-reducing conditions. The membrane detection was done by using both Amersham Imager 600 (Thermo Fisher Scientific, Inc., Toronto, ON, Canada) and autoradiography films. The digital images were analyzed using Image J win-64 software (University of Wisconsin-Madison, Madison, WI, USA). The band intensities of the protein were normalized to that of the tubulin to compare the level of expression of the protein of interest.

### 2.7. Statistical Analysis

All data were presented as mean ± SEM. Normality of the data and homogeneity of variances were determined by the Shapiro–Wilk and Bartlett tests, respectively. Because we euthanized different rats at each time point, statistical differences between groups were determined using Two-Way ANOVA and Bonferroni test (to assess treatment and time-specific changes) using GraphPad Prism 5 (GraphPad Software, Inc., San Diego, CA, USA). The Mann–Whitney test was used to determine statistical differences in sperm motility and DNA oxidation among groups. Differences with a *p*-value of ≤0.05 were considered significant.

## 3. Results

### 3.1. Low Sperm Motility and High DNA Oxidation Suggest Compromised Sperm Quality Due to Oxidative Stress

**Figure 1:**
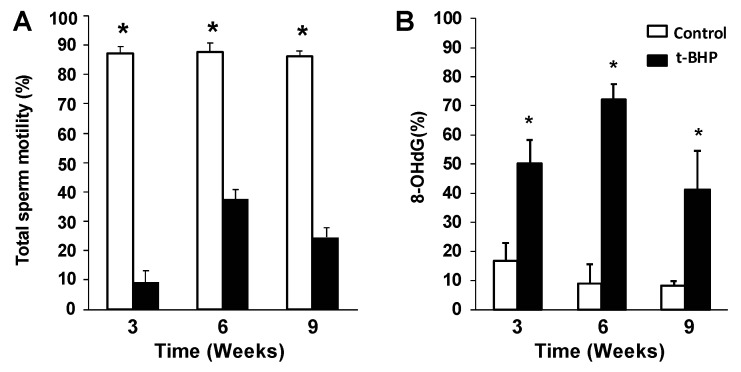
Impairment of sperm quality due to t-BHP treatment in male rats. (**A**) Sperm motility and (**B**) sperm DNA oxidation determined by 8-deoxyguanosine (8-OHdG) levels. The results are expressed as mean ± SEM. * Means higher than the other group at the same time point (*p* ≤ 0.05; Mann–Whitney test, *n* = 4).

Spermatozoa from cauda epididymis were collected and analyzed 3 weeks, 6 weeks and 9 weeks after the end of the t-BHP treatment to determine whether there were damages due to the treatment during the late, middle and early spermatogenesis, respectively. Spermatozoa from t-BHP-treated animals showed a significant reduction of their motility at all times compared to those sperm from the control group. Moreover, we observed significantly higher DNA oxidation levels in spermatozoa from treated rats compared to controls (Figure 1).

### 3.2. Lipid Peroxidation Increased in Caput and Cauda Epididymis of t-BHP Treated Male Rats

**Figure 2:**
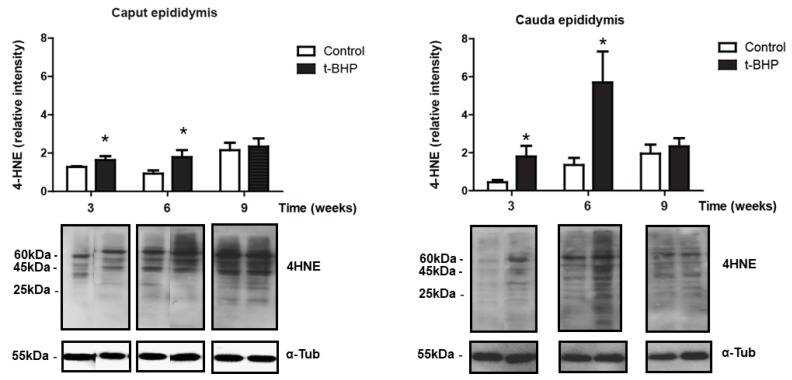
Lipid peroxidation (determined by 4-HNE levels) increased in caput and cauda epididymis in t-BHP compared to control male rats. The results of relative intensities (upper panels) are expressed as mean ± SEM. The blots presented are representative of experiments with 4 different rats. Some lanes showing protein bands have been pasted but belong to the same blot and have the same film exposure. * Means higher than the other group at the same time point (*p* ≤ 0.05; Two-way ANOVA and Bonferroni post-hoc test, *n* = 4).

We determined oxidative damage on lipids by detecting 4-hydroxynonenal (4-HNE), a known marker of lipid peroxidation. We observed multiple bands detected by the anti-4HNE antibody, suggesting that different proteins contain the 4-HNE adduct in the tissues analyzed (Figure 2). A significant increase of 4-HNE levels was observed in both the caput and cauda epididymis of treated rats at 3 weeks and 6 weeks after the end of the t-BHP treatment compared to controls. Interestingly, the levels of 4-HNE at 9 weeks after the end of treatment were similar in the caput epididymis when comparing treated and control rats but were significantly higher than those seen in the control group at 6 weeks. In cauda epididymis, the 4-HNE levels returned to control values at 9 weeks after treatment. Noteworthily, the levels of lipid peroxidation at 9 weeks was higher in caput compared to cauda epididymis, suggesting an early dysregulation of the antioxidant response in the caput epididymis. 

### 3.3. PRDX1 and PRDX6 Are Differentially Upregulated in Caput and Cauda Epididymis at Different Time Points

**Figure 3:**
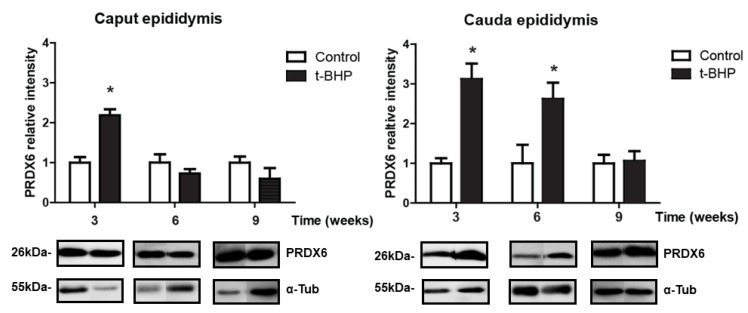
Peroxiredoxin 6 (PRDX6) expression in caput and cauda epididymis of control and t-BPH-treated male rats. The results of relative intensities (upper panels) are expressed as mean ± SEM. The blots presented are representative of experiments with 4 different rats. Some lanes showing protein bands have been pasted but belong to the same blot and have the same film exposure. * Means higher than the other group at the same time point (*p* ≤ 0.05; Two-way ANOVA, *n* = 4).

**Figure 4:**
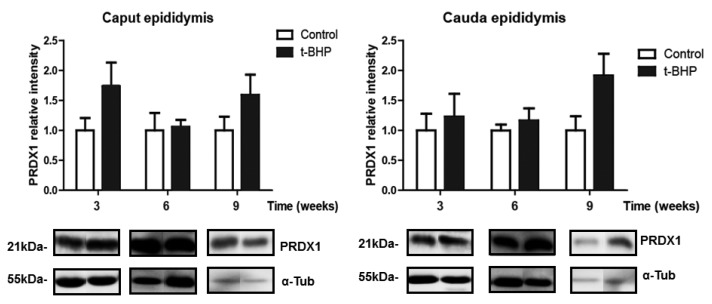
Peroxiredoxin 1 (PRDX1) expression in caput and cauda epididymis of control and *t*-BPH-treated male rats. The results of relative intensities (upper panels) are expressed as mean ± SEM, (*p* > 0.05; Two-way ANOVA and Bonferroni post-test, *n* = 4). The blots presented are representative of experiments with 4 different rats. Some lanes showing protein bands have been pasted but belong to the same blot and have the same film exposure.

We assessed the expression levels of PRDX1 and PRDX6 in rat epididymis, and we observed a significant increase of PRDX6 in caput epididymis at 3 weeks and in cauda epididymis at 3 and 6 weeks post-treatment (Figure 3). We also found a trend of increase in PRDX1 expression levels in caput epididymis at week 3 and 9, and in cauda epididymis at week 9, but these increases were not significant (Figure 4). 

### 3.4. Catalase Expression Shows Trends of Increase, with Significant Individual Variation in the Epididymis

**Figure 5:**
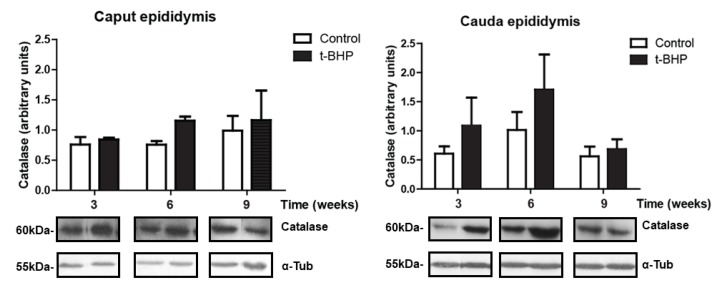
Catalase expression in caput and cauda epididymis of control and t-BPH-treated male rats. The results of relative intensities (upper panels) are expressed as mean ± SEM, (*p* > 0.05; Two-way ANOVA and Bonferroni post-test, *n* = 4). The blots presented are representative of experiments with 4 different rats. Some lanes showing protein bands have been pasted but belong to the same blot and have the same film exposure.

Catalase expression levels were determined in rat epididymis from control and treated rats. Although not significant, we observed a trend of increase in treated rats compared to control at 3 and 6 weeks in cauda epididymis (Figure 5). Caput epididymis did not show upregulation of catalase at any time point.

### 3.5. PRDXs, Catalase and Thioredoxin Expression Levels and Lipid Peroxidation Are Similar in Testis Despite the t-BHP Treatment

**Figure 6:**
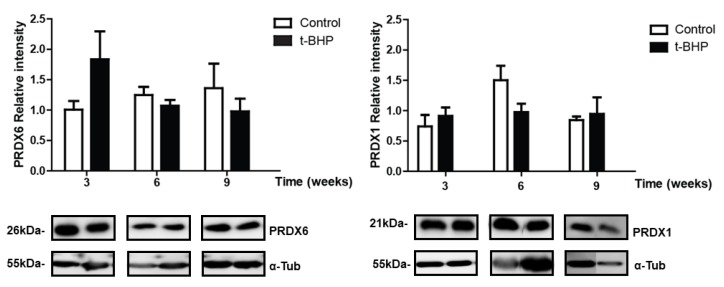
Peroxiredoxin 1 (PRDX1) and 6 (PRDX6) expression in testis of control and t-BPH-treated male rats. The results of relative intensities (upper panels) are expressed as mean ± SEM, (*p* > 0.05; Two-way ANOVA and Bonferroni post-test, *n* = 4). The blots presented are representative of experiments with 4 different rats. Some lanes showing protein bands have been pasted but belong to the same blot and have the same film exposure.

**Figure 7:**
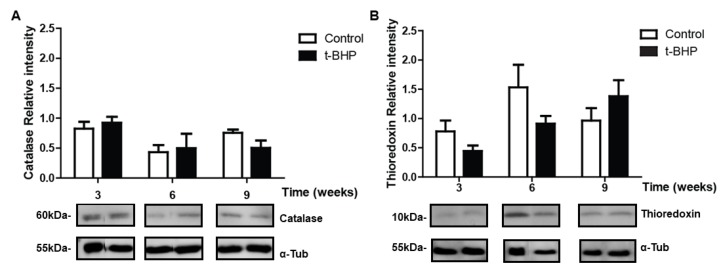
Catalase (**A**) and thioredoxin (**B**) expression in testis of control and t-BPH-treated male rats. The results of relative intensities (upper panels) are expressed as mean ± SEM, (*p* > 0.05; Two-way ANOVA and Bonferroni post-test, *n* = 4). The blots presented are representative of experiments with 4 different rats. Some lanes showing protein bands have been pasted but belong to the same blot and have the same film exposure.

No significant difference was observed between the control and treated groups at any time points for PRDX1, PRDX6, catalase or TRX-1 (Figure 6 and Figure 7).

**Figure 8:**
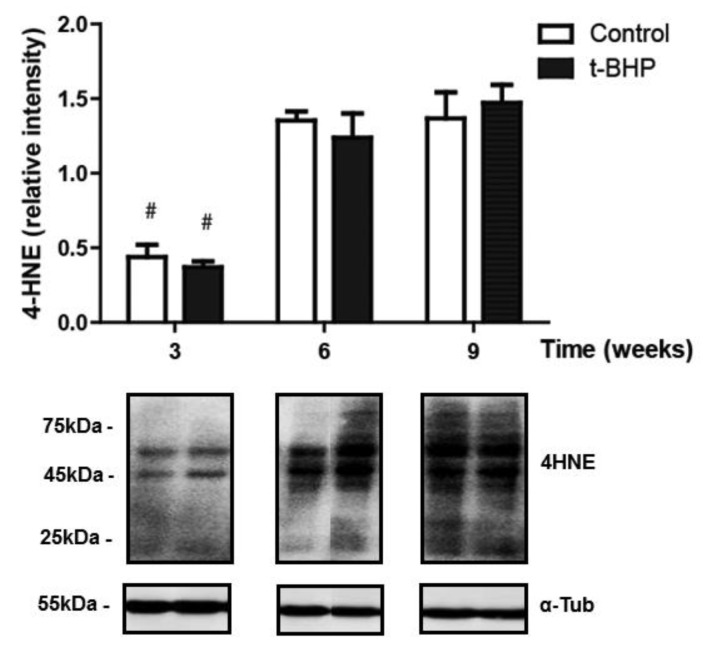
Lipid peroxidation in the testis of control and t-BPH-treated male rats. The results of relative intensities (upper panels) are expressed as mean ± SEM. The blots presented are representative of experiments with 4 different rats. Lanes showing protein bands have been pasted but belong to the same blot and have the same film exposure. ^#^ Means lower than all other groups, (*p* ≤ 0.05; Two-way ANOVA and Bonferroni post-test, *n* = 4).

The levels of 4-HNE in testis were similar in control and treated rats, suggesting that the oxidative stress generated by the treatment was well tolerated by the testis (Figure 8). Noteworthy, the levels of 4-HNE increased in testis from both control and treated rats at 6 and 9 weeks, suggesting that there is time dependency in the levels of lipid peroxidation in this organ. 

### 3.6. Reproductive Organs Weight, Spermatogenesis, and Sperm Production Were Not Affected by the t-BHP Treatment

**Figure 9:**
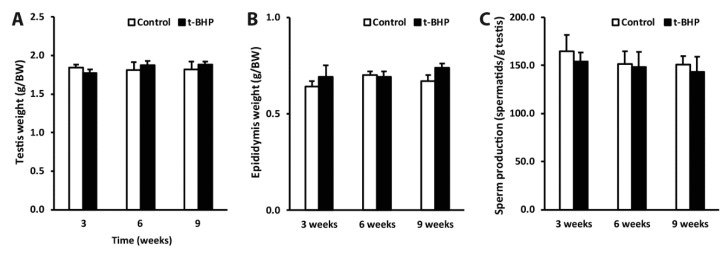
Testis and epididymis weight (A and B) and sperm production (C). (*n* = 4, Two-way ANOVA, *p* > 0.05).

To determine whether the damages observed in epididymal spermatozoa may have originated due to impairment of spermatogenesis by t-BHP treatment, we analyzed testis sections from control and treated groups to identify the different stages of spermatogenesis, compared the reproductive organs weight and sperm production in the experimental groups. We did not observe differences in body and organ weights between the treated and control rats at any time point (Figure 9 and Supplementary Materials Table S1). 

**Figure 10:**
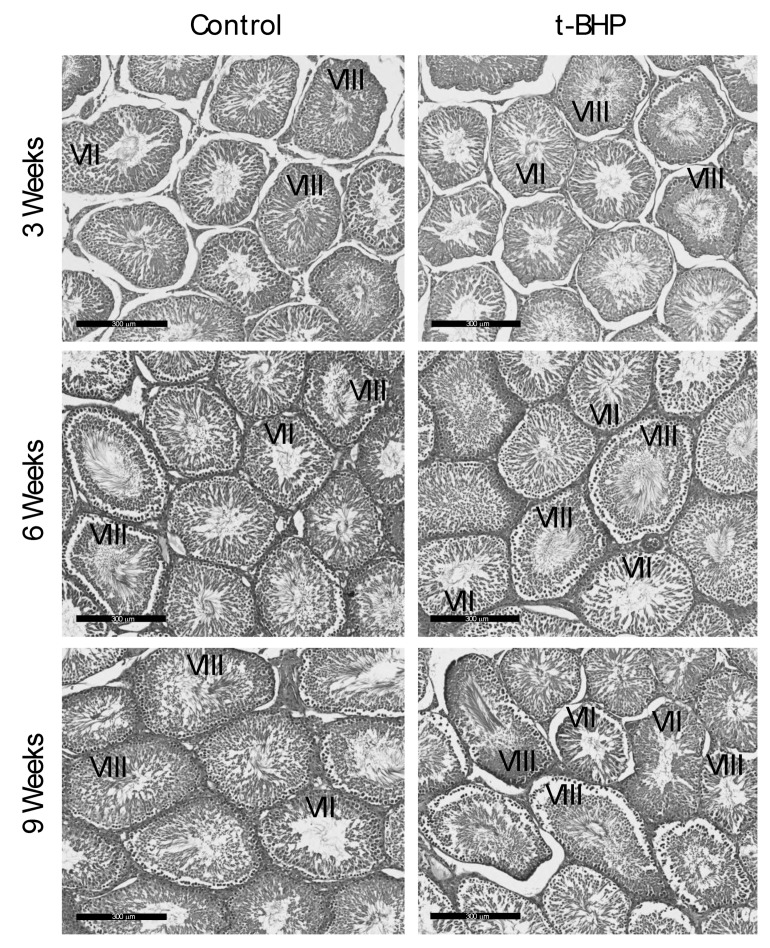
Histological analysis of testes from control and t-BHP-treated male rats. Testis sections were stained with hematoxylin and eosin to evaluate spermatogenesis. All testis sections displayed normal spermatogenesis (Stages VII and VIII showing elongating spermatids and spermatozoa in the lumen of the seminiferous tubules, respectively), *n* = 4. Bar = 300 μm.

The analysis of testis sections revealed that spermatogenesis proceeded normally in both control and treated rats during the 9 weeks after the end of treatment (Figure 10) that spermatogonia are transformed into spermatozoa [25]. We identified all the stages of the spermatogenesis in the rat, including the stages VII and VIII that contained elongating spermatids and fully formed spermatozoa, respectively in the luminal edge of the seminiferous epithelium, indicating active sperm production by the testes. As shown in Figure 10, some seminiferous tubules contain spermatozoa in the lumen (stage VIII), be ready to be spermiated to enter the epididymis. In addition, Sertoli and Leydig cells were morphologically normal.

## 4. Discussion

The present study shows for the first time the long-term adverse effect of t-BHP induced in vivo oxidative stress on rat spermatozoa and epididymis and the differential expression of antioxidant enzymes that fight against hydroperoxides in epididymis and testis. The reduced motility and high DNA damage observed in rat cauda epididymal spermatozoa collected at 3 weeks, 6 weeks and 9 weeks after the end of t-BHP treatment indicates the long-lasting adverse effects of oxidative stress on sperm quality. This result was unexpected since after one cycle of spermatogenesis (9 weeks in the rat) [25], new spermatozoa were produced from spermatogonia and these spermatozoa were those present in the epididymis at the time of collection [28]. Indeed, these collected spermatozoa showed significant DNA oxidative and impaired motility (Figure 1). These findings suggest that the balance between ROS and the antioxidant system has been compromised in the testis and or epididymis after the t-BHP-induced oxidative stress. 

Based on the time of the collections and the length and stages of rat spermatogenesis, the collected rat spermatozoa were spermatids, spermatocytes or spermatogonia at the time that the rat was exposed to the t-BHP treatment [28]. When spermatozoa leave the testis, they enter into the epididymis to undergo their maturation before ejaculation [21]. Since the treatment has struck both testis and epididymis, the detrimental effects observed in the spermatozoa retrieved form the cauda epididymis could be a consequence of a direct impact of oxidative stress on the germ cells during spermatogenesis or due to the detrimental and persistent effects of high levels of ROS in the epididymal epithelium that impairs the proper epididymal maturation of spermatozoa. 

In the epididymis, we expected that the increased expression of antioxidant enzymes would reduce the oxidative damage in spermatozoa because of the transfer of antioxidant enzymes from the epididymis to spermatozoa through the secretion of epididymosomes [23]. However, high 4-HNE levels in caput and cauda epididymis are an indication of developing lipid peroxidation (Figure 2) and suggest that the epididymal epithelium itself was damaged by the oxidative stress and therefore unable to protect spermatozoa during their maturation. The upregulation of PRDX6 and the trend of increase of PRDX1 and catalase expression in epididymis were not sufficient to scavenge the excessive ROS and restore the healthy cellular environment for a normal sperm epididymal maturation. We observed a similar response by the epididymis of rats treated for two weeks with t-BHP, with an upregulation of PRDX1 and PRDX6 but not of catalase [18].

Contrarily to what was observed in the epididymis, there were no significant differences in the levels of antioxidant enzyme expression and lipid peroxidation in the testes of treated rats compared to controls. Furthermore, the histological analysis and the testis weight and spermatid count indicated that there was no disruption of spermatogenesis. These findings suggest that there was no evidence of oxidative stress during spermatogenesis that could damage the collected spermatozoa at the different end points. Testicular spermatozoa have lower levels of DNA damage compared to the ejaculated counterparts [29,30]. Moreover, the fact that testicular spermatozoa from infertile men with obstructive azoospermia have low levels of DNA oxidation that do not interfere with the formation of an embryo by intracytoplasmic sperm injection suggests that the level of DNA oxidation in testicular sperm is not detrimental for male fertility [31]. It is plausible that the testicular spermatozoa are more resistant to oxidative stress compared to epididymal spermatozoa that flow freely in the lumen because the developing spermatozoa are guarded by the Sertoli cells that provide nutrients [32,33] and antioxidant protection through SOD, GSTs, GPXs, and PRDXs [34,35,36]. 

During epididymal maturation, the sperm chromatin is further compacted and could be exposed to oxidative stress generated by different conditions. Thus, it is of paramount importance that the epididymal epithelium protect the maturing spermatozoon against oxidative stress. The finding that PRDX6 expression levels are high when lipid peroxidation (measured by 4-HNE levels) are increased in cauda epididymis of treated rats, collected at week 3 and week 6, while these levels return to those of controls at week 9, indicate that PRDX6 is an essential component of the antioxidant response in the epididymis. 

In a previous study, we challenged rats with the same t-BHP treatment and found that caudal epididymal spermatozoa collected 24 h after the end of the treatment had increased DNA oxidation, and reduced motility [18]. These findings indicated the negative effect of in vivo oxidative stress exclusively on epididymal maturation. We observed similar damages in mouse lacking PRDX6, a condition that generates an in vivo oxidative stress and is associated with male infertility [16]. The *Prdx6^−/−^* spermatozoa have low motility and high levels of DNA oxidation and lipid peroxidation. The *Prdx6^−/−^* spermatozoa also had higher percentages of cytoplasmic droplet retention compared to wild-type cells [16], an indication of abnormal epididymal maturation. During the epididymal transit, spermatozoa shed the residual cytoplasm; thus, an increase in spermatozoa carrying cytoplasmic droplets is an indication of abnormal epididymal maturation [37]. 

PRDX6 is a unique antioxidant enzyme as it is the only antioxidant enzyme known to date, with calcium-independent phospholipase A_2_ (iPLA_2_) [38] and lysophosphatidylcholine acyl transferase activities (LCAT) [39]. Both PRDX6 iPLA_2_ and LCTAT activities are essential to remove and replace the oxidized phospholipids with newly synthesized phospholipids [39,40]. The epididymis increases PRDX6 in response to the oxidative damage caused by ROS to try to repair oxidized membrane lipids. A recovery of lipid peroxidation to the control level was observed in epididymis and spermatozoa at week 9, suggesting that PRDX6 repaired the damaged lipid membranes in this organ. This tendency was found in cauda epididymis but not in caput epididymis. We previously indicated that PRDX6 plays a crucial role in protecting both the epithelium and the spermatozoa in the cauda epididymis segment specifically [18]. Noteworthily, the higher levels of 4-HNE found in caput compared to cauda epididymis at week 9 suggest a differential capacity of antioxidant response in the different parts of the epididymis.

Although some repair of oxidative damage such as lipid peroxidation was observed in the caput and cauda epididymis at 6 and 9 weeks (Figure 2) and in spermatozoa at 9 weeks (Figure 1), epididymal spermatozoa had significant DNA oxidation at all time points. There is a possibility that the oxidative damage sperm DNA is a consequence of the impact of the treatment on the testis. Treated rats had similar sperm production than controls; their testes did not show long-lasting oxidative damage as the epididymis and were morphologically similar to control testis. Thus, it is less likely that the sperm DNA oxidation is due to problems during spermatogenesis. However, we cannot exclude the possibility that some of the damage observed in the sperm DNA may occur during the formation of spermatozoa in the testis. The oxidative stress generated by t-BHP altered the expression of miRNAs involved in the antioxidant response and spermatogenesis in mouse testis [41]. Although we did not see significant changes in spermatogenesis and the antioxidant response appears to be intact in rat testis, there is a possibility for the disruption of molecular mechanisms driven by miRNAs or epigenetic changes that can be associated with the permanent sperm DNA damage observed in this study. Further studies are required to rule out these possibilities.

While we presented evidence that the antioxidant response of the rat epididymis against oxidative stress is altered and may explain the poor quality of spermatozoa observed in this study, multiple factors could contribute to the persistent DNA oxidation observed in spermatozoa from t-BHP-treated rats. Sperm chromatin is a highly organized structure that differs from that of the somatic cells. Protamines replace histones during spermatogenesis, allowing the chromatin to tightly compact [42,43]. During epididymal transit, protamines become thiol oxidized and make disulfide bridges among them, thus, making the sperm chromatin more compacted [43]. Low mature protamine to protamine precursor ratio has been found in infertile patients and is correlated with high DNA damage, suggesting that chromatin compaction is critical for the protection of sperm DNA [43,44]. In our study, the persistent DNA damage observed in rat spermatozoa could be due to alterations of the sperm chromatin structure that interfered with normal sperm chromatin compaction, thus making sperm DNA more susceptible to the oxidative stress seen after 9 weeks of the end of treatment. Permanent oxidative stress in the male reproductive system as the one observed in *Prdx6^−/−^* male mice leads to changes in the sperm chromatin with increased DNA oxidation and lower protamination (amounts of protamines) and DNA compaction compared to the wild-types controls [16]. Exposure of male rats to the chemotherapeutic agent cyclophosphamide, known to produce ROS as part of the mechanism of action, decreased the level of protamination and subsequently increased DNA damage of rat spermatozoa [45]. 

Similar long-lasting damages as those found in this study were observed in testicular cancer survivors who underwent chemotherapy with cisplatin and bleomycin, two drugs that generate high levels of ROS in cells exposed to them [46,47]. Cancer patients treated with polychemotherapy, including ROS-generating compounds, have high levels of lipid peroxides in blood, indicating the establishment of oxidative stress due to the treatment [48]. Spermatozoa from testicular cancer survivors displayed high DNA damage and low DNA compaction up to two and one years, respectively, after the end of chemotherapy [49,50]. With this significant clinical relevance, the present study provides insight into the understanding of the long-term effect of oxidative stress, a condition often seen in male infertility [2,3].

The molecular mechanism behind the long-term, lasting oxidative stress observed in this study is yet to be determined. A potential candidate is the dysregulation of mitochondria due to high levels of 4-HNE. We hypothesize that the high oxidative stress due to t-BHP promoted significant 4-HNE levels that impaired mitochondrial proteins leading to dysregulation of this organelle. We observed that the inhibition of PRDX6 iPLA_2_ activity by MJ33 increased the levels of 4-HNE and impaired the sperm mitochondrial membrane potential, leading to the generation of oxidative stress and the oxidation of the DNA in human spermatozoa [15]. 4-HNE is capable of inducing mutations of the mitochondrial DNA and form adducts with mitochondrial proteins that lead to mitochondrial dysfunction [51]. Further studies are needed to elucidate the molecular mechanisms behind the alterations in spermatozoa and epididymis observed in the present study.

## 5. Conclusions

In conclusion, we reported the unexpected long-term effects of t-BHP treatment in the male rat reproductive system that impairs sperm quality after one complete cycle of spermatogenesis. The epididymis, in contrast to the testis, was primarily affected by the treatment displaying markers of oxidative stress such as high levels of 4-HNE up to 9 weeks after the end of the treatment. An antioxidant response by the upregulation of PRDX6 and possibly PRDX1 and catalase attempt to correct the oxidative stress in the epididymis results in the decrease of lipid peroxidation at 9 weeks in cauda epididymis, but it appears not to be sufficient to repair the oxidative damage observed in spermatozoa. Further studies will be needed to elucidate the molecular mechanism that generates long-term oxidative stress that impairs sperm quality and fertility. These studies are relevant since many conditions such as diseases (i.e., cancer, diabetes), drugs and even lifestyles (i.e., smoking) generate chronic oxidative stress that impacts male fertility.

## Supplementary Materials

The following are available online at https://www.mdpi.com/2076-3921/9/2/170/s1, Table S1: Body and reproductive organs weight and sperm production.
