# Supplementary material for: Long-Term Adverse Effects of Oxidative Stress on Rat Epididymis and Spermatozoa
Source: Antioxidants (Basel). 2020 Feb 19;9(2):170. doi: 10.3390/antiox9020170 (PMC7070312; doi:10.3390/antiox9020170)
Supplement: Supplementary file 1 [file antioxidants-09-00170-s001.pdf]

Long-Term Adverse Effects of Oxidative Stress on Rat Epididymis and Spermatozoa

Pei You Wu, Eleonora Scarlata and Cristian O'Flaherty

Supplementary table S1 - Body and reproductive organs weight and sperm production

		**Body weight (g)**	**Testis (g)**	**Epididymis (g)**	**Ventral prostate (g)**	**Seminal vesicles (g)**	**Coagulating glands (g)**	**Sperm production ($x10^6$ spermatids/g testis)**
3 weeks	Control	511.75±10.70	1.84±0.04	0.64±0.03	0.82±0.04	0.82±0.08	0.23±0.04	164.50±17.00
	tert-BHP	542.00±10.86	1.77±0.05	0.69±0.06	0.73±0.07	0.65±0.06	0.18±0.03	154.00±9.50
6 weeks	Control	577.00±10.86	1.81±0.10	0.70±0.02	0.81±0.06	0.62±0.08	0.21±0.03	151.75±12.90
	tert-BHP	610.50±11.14	1.87±0.06	0.69±03	0.78±0.03	0.54±0.06	0.23±0.04	148.63±15.40
9 weeks	Control	556.75±24.24	1.82±0.10	0.67±0.03	0.75±0.04	0.71±0.11	0.22±0.04	150.68±9.00
	tert-BHP	666.50±18.77	1.88±0.04	0.74±0.02	0.71±0.05	0.66±0.05	0.21±0.01	143.43±15.6

Body and organ weights were not different between the groups (n = 4, Two-Way ANOVA, $p < 0.05$).
